# Safety and feasibility of transjugular intrahepatic portosystemic shunt in elderly patients with liver cirrhosis and refractory ascites

**DOI:** 10.1371/journal.pone.0235199

**Published:** 2020-06-25

**Authors:** Lena Stockhoff, Marie Schultalbers, Tammo L. Tergast, Jan B. Hinrichs, Svetlana Gerbel, Timo C. Meine, Michael P. Manns, Nicolas Simon, Markus Cornberg, Bernhard C. Meyer, Benjamin Maasoumy

**Affiliations:** 1 Department of Gastroenterology, Hepatology and Endocrinology, Hannover Medical School, Hannover, Germany; 2 Institute for Diagnostic and Interventional Radiology, Hannover Medical School, Hannover, Germany; 3 Centre for Information Management (ZIMt), Hannover Medical School, Hannover, Germany; 4 Centre for Individualised Infection Medicine (CIIM), c/o CRC Hannover, Hannover, Germany; 5 German Centre for Infection Research (Deutsches Zentrum für Infektions-forschung DZIF), Partner-site Hannover-Braunschweig, Hannover, Germany; Medizinische Fakultat der RWTH Aachen, GERMANY

## Abstract

**Background & aims:**

The management of patients with refractory ascites (RA) is challenging, particularly at higher age. Transjugular intrahepatic portosystemic shunt (TIPS) is an established treatment for RA, but safety data in elderly patients are rare. Our aim was to evaluate the safety and feasibility of TIPS in elderly patients with RA.

**Methods:**

Overall, 160 consecutive cirrhotic patients receiving a TIPS for RA at Hannover Medical School between 2012 and 2018 were considered for this retrospective analysis. Periinterventional complications such as acute-on-chronic liver failure (ACLF) as well as survival were compared between patients <65 and ≥65 years. Propensity score matching was conducted to match elderly TIPS patients and patients treated with paracentesis.

**Results:**

A number of 53 out of the 160 patients were ≥65 years (33%). Periinterventional course in those ≥65 years appeared to be slightly more complicated than in <65 years as reflected by a significantly longer hospital stay (p = 0.030) and more ACLF-episodes (21% vs. 9%; p = 0.044). 28-day mortality was similar between both groups (p = 0.350), whereas survival of the younger patients was significantly higher at 90 days (p = 0.029) and numerically higher at 1 year (p = 0.171). In the multivariate analysis age ≥65 years remained an independent predictor for 90-day mortality (HR: 2.58; p = 0.028), while it was not associated with 28-day and 1-year survival. Importantly, after matching for potential confounders 1-year survival was similar in elderly patients if treated with TIPS or paracentesis (p = 0.419).

**Conclusions:**

TIPS placement in elderly patients with RA appears to be slightly more complicated compared to younger individuals, but overall feasible and at least not inferior to paracentesis.

## Introduction

The clinical management of patients with liver cirrhosis and refractory ascites (RA) is challenging. Mortality of these patients is markedly high reaching up to 50% within 1 year [1,2]. Currently, the only curative treatment option for RA is liver transplantation, but at present the availability of donor organs is highly limited and in case of patients at higher age transplantation is often restricted. An alternative established treatment option for patients with RA is the insertion of a transjugular intrahepatic portosystemic shunt (TIPS).

TIPS placement results in an immediate reduction of portal hypertension. According to various studies, TIPS insertion shows the tendency to improve survival as compared to large volume paracentesis [3,4]. However, TIPS placement bears the risk of developing hepatic encephalopathy (HE) or aggravation of the hyperdynamic circulatory state, which might result in short-term cardiac failure [5]. Shunt creation can even be harmful, particularly in patients with most advanced stages of liver disease [6]. Thus, a careful selection of patients is crucial. However, there are many controversies regarding the definite selection criteria for TIPS insertion in patients with RA. According to current EASL guidelines, TIPS is not recommended in patients suffering from recurrent or overt HE, heart failure, active infection, severe liver dysfunction or pulmonary hypertension. Of note, the parameter age is not particularly mentioned [4]. However, TIPS insertion in elderly patients is supposed to be performed with great caution, since it might increase the risk for HE in patients of higher age [7]. In fact, higher age is even considered as a contraindication in many centers [8].

So far, safety data of TIPS in the older population are scarce and age as a relative or even absolute contraindication for TIPS placement–especially for the recently used covered TIPS–has not been properly addressed. In most randomized controlled trials, which were also the basis of various meta-analyses, patients of higher age were excluded [6,9–11]. Only a very few retrospective studies directly evaluated the applicability of TIPS in elderly patients, but their results are controversial and in none of these an appropriate paracentesis control group was included. Thus, no decisive conclusions can be drawn from the current literature with respect to the applicability of TIPS in elderly patients. As the proportion of elderly patients with liver cirrhosis has been increasing [12], the concern of applicability and safety of TIPS placement in patients of higher age becomes progressively important these days.

The aim of this study was to evaluate the feasibility and safety of TIPS placement in elderly patients with decompensated liver cirrhosis and RA.

## Materials and methods

### TIPS cohort

**Fig 1:**
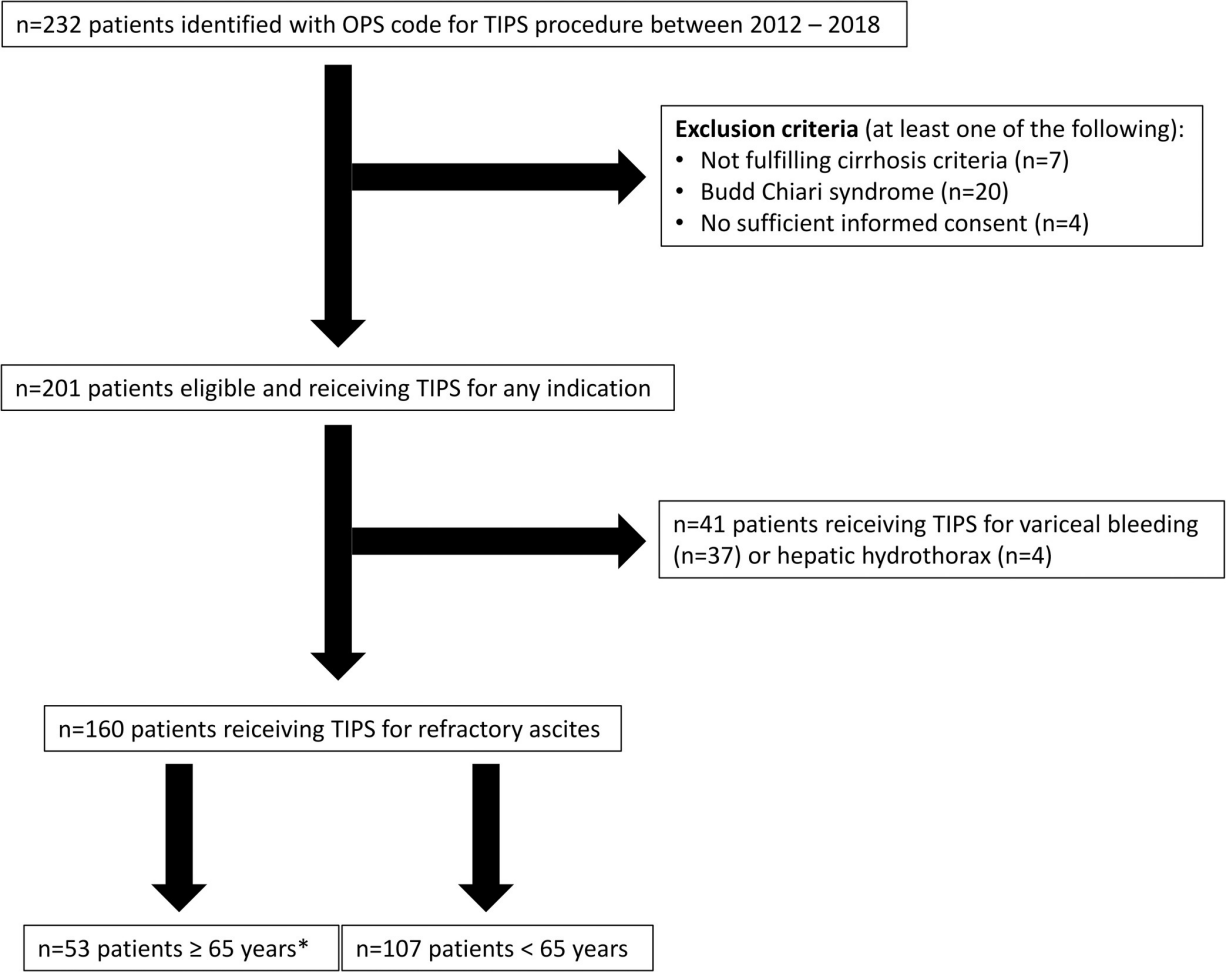
Patient selection algorithm and distribution of patients‘ age. * n = 14 patients of the elderly subgroup were ≥75 years. Abbreviations: OPS: operation and procedure code.

All consecutive patients receiving a TIPS between January 2012 and December 2018 at Hannover Medical School were automatically identified by the Enterprise Clinical Research Data Warehouse comprising clinical data of >2.2 million patients. The German operation and procedure code encoding the TIPS surgical procedure was used to search for TIPS patients (Fig 1). Subsequently, all patients without sufficient informed consent, without manifest criteria of liver cirrhosis as well as patients with Budd Chiari syndrome were excluded. In order to be able to adequately match TIPS patients with paracentesis patients, we restricted the analysis to patients with RA. The elderly population was defined as patients ≥65 years at TIPS insertion, because this is a widely accepted age limit indicating a more careful evaluation before transplantation and is most commonly used in clinical trials [13]. Overall, 160 patients met the inclusion criteria.

### Ascites cohort

Patients eligible for the paracentesis group were recruited from the well-defined Hannover Ascites Cohort comprising of >600 patients with decompensated liver cirrhosis and ascites [14,15]. Albumin substitution was used after large volume paracentesis at our center in concordance with current guidelines [4]. Only patients ≥65 years suffering from RA were selected for the matching procedure. In order to minimize bias, patients fulfilling any overt TIPS contraindication such as pulmonary hypertension, hepatocellular carcinoma at baseline, severe myocardial dysfunction, HE ≥ grade 2 or chronic HE were excluded [4]. In addition, patients with acute clinical deterioration, Budd Chiari syndrome and/or ongoing uncontrolled infection were excluded. Overall, 85 elderly patients were eligible for the propensity score matching.

### Data assessment

The clinical, laboratory and TIPS procedure related data were extracted from the patients’ medical records. The data were accessed between July 2019 and March 2020. Baseline was set at the day of TIPS insertion or first paracentesis at Hannover Medical School, respectively. Laboratory data that were closest to baseline were considered for the analysis. Refractory/recurrent ascites was defined as resistant/intractable to diuretics in maximal concentrations and/or recurrence of ascites 3 times in a period of 1 year [1]. Insufficient ascites control during follow up was defined as ascites requiring large volume paracentesis and/or at least grade 2 in ultrasound measurements within a time span of 3 to 6 months after TIPS insertion. Infections were diagnosed based on clinical symptoms, laboratory values and/or the estimation of the treating physician [16]. SBP was diagnosed in concordance with local standards and German guidelines if ascitic fluid contains ≥250 polymorphonuclear leukocytes or ≥500 nucleus containing cells per μL [17]. HE was classified according to West Haven criteria [3] and acute kidney injury (AKI) was defined based on the recommendations of the International Club of Ascites [18]. Acute-on-chronic liver failure (ACLF) was diagnosed as reported in the EASL guidelines [19], whereas terlipressin for treatment of hepatorenal syndrome in the absence of hypotension was not considered as circulatory failure.

### TIPS placement

TIPS placement was performed by clinically experienced interventional radiologists (BCM, JBH and TCM) according to the institutional standard operating procedure [20,21]. In all patients covered stents (Viatorr^®^, Gore, Flagstaff, Arizona, AZ, USA) with a prosthesis diameter of 8 mm (n = 151) or 10 mm (n = 9) or were used.

### Study design

Primary endpoint of the study was mortality 28 days, 90 days and 1 year after TIPS insertion. Patients were censored if they underwent liver transplantation (n = 7 TIPS patients and no patient from the paracentesis cohort) or end of follow-up. A multivariate model adjusting for degree of portal hypertension (portosystemic pressure gradient (PSG)), severity of liver disease (MELD), sex and etiology of liver cirrhosis was applied. Secondary endpoints included periinterventional complications such as occurrence of infections, HE, AKI and ACLF during hospital stay after TIPS insertion and duration of hospital stay as well as ascites control and changes in the serum creatinine levels after TIPS insertion.

In order to compare TIPS with paracentesis in cirrhotic elderly suffering from RA, an 1:1 propensity score matching was used [22–24]. Matching covariates were MELD, sex, age, bilirubin, platelet count and sodium. Subsequently, mortality 28 days, 90 days and 1 year after baseline was analyzed in the matched cohort. Baseline was defined as TIPS insertion or time of first paracentesis, respectively.

### Statistics

All statistical analyses were performed using SPSS (IBM SPSS Statistics, Versions 25+26), R Version 3.3.3 (packages ‘MatchIt’ [25], ‘RItools’ [26], and ‘cem’ [27]) and Microsoft Excel 2010. Continuous variables are presented as median with interquartile range (IQR) and compared using the Mann-Whitney-U-Test for unpaired data or the Wilcoxon signed-rank test for paired data, respectively. Categorical variables are shown as numbers with percentages and compared using the chi-squared test or Fisher’s exact test. Paired categorical variables were compared using the McNemar test. Survival was analyzed with the log-rank test as well as the Breslow test and visualized using Kaplan-Meier curves. To adjust for potential confounders, uni- and multivariate Cox regression analysis (backwards stepwise regression) was performed including all clinically relevant factors tested in the univariate model. In case of missing values within the Cox regression model, the case was excluded for this particular analysis. A value of p<0.05 was considered statistically significant.

Propensity score matching was conducted using an 1:1 nearest neighbor matching procedure based on the greedy matching algorithm [28]. This algorithm improves imbalance between the groups by making best matches first and then taking the next best matches until no more matches can be made in a hierarchical sequence. Uni- and multivariate testing of model adequacy, as well as visual inspection of the distribution of standardized mean differences (SMD) of the covariates (S1 Fig and S1 Table) was used to validate the achieved reasonable balance between the groups.

### Ethics

This study was approved by the local ethics committee of Hannover Medical School and followed the principles of the Declaration of Helsinki. All patients included in the analysis provided written informed consent for the usage of their clinical data for scientific purposes.

## Results

### Baseline characteristics of patients receiving a TIPS for refractory ascites

A number of 160 TIPS patients were included in this study with a median age of 59 years and a median MELD of 12.6 (Table 1). 56% of the patients were males. Hemodynamic success after TIPS insertion, which was defined as a final PSG ≤12 mmHg, was achieved in 99.4% of the patients. The median preinterventional PSG was 16.2 mmHg and the median postinterventional PSG was 5.9 mmHg resulting in a median PSG reduction of 63.6%. A number of 53 patients (33%) were ≥65 years and 107 patients (67%) were <65 years. In the majority of patients (61%) the etiology of cirrhosis was alcohol-related. In the younger patients the amount of alcohol-related liver disease was significantly higher (66% vs. 49%; p = 0.035). Of note, the majority of baseline laboratory values, including MELD and platelet count as well as sex were not different between the young and the elderly. However, the final PSG was significantly lower in patients ≥65 years (p = 0.037) resulting in a greater PSG reduction (p = 0.050). Furthermore, in the elderly patients creatinine levels were significantly higher (p = 0.001), whereas the INR at baseline was significantly lower (p = 0.033) (Table 1).

**Table 1:** Baseline characteristics of TIPS patients.

	All patients	≥ 65 years	< 65 years	*P* value
Patients (n, %)	160 (100)	53 (33)	107 (67)	
Age (y)	59 (52–68)	70.0 (68–75)	55.0 (49–59)	< .001
Male/female (n, %)	89 (56)/ 71 (44)	30 (57)/ 23 (43)	59 (55)/ 48 (45)	.861
Etiology of cirrhosis*				
Viral (n, %)	19 (12)	7 (13)	12 (11)	.796
Alcohol (n, %)	97 (61)	26 (49)	71 (66)	.035
NASH (n, %)	12 (8)	6 (11)	6 (6)	.214
Other (n, %)	36 (23)	14 (27)	22 (21)	.404
PSG before TIPS (mmHg)	16.2 (13.2–19.9)	16.2 (12.5–20.6)	16.2 (13.8–19.1)	.891
PSG after TIPS (mmHg)	5.9 (4.4–7.4)	5.1 (3.7–7.2)	5.9 (4.4–8.0)	.037
% reduction of PSG	63.6 (54.1–72.0)	65.2 (56.0–77.2)	62.0 (52.4–70.7)	.050
Stent diameter				
8 mm (n, %)	151 (94)	50 (94)	101 (94)	1.000
10 mm (n, %)	9 (6)	3 (6)	6 (6)	1.000
MELD	12.6 (10.1–15.6)	12.6 (10.8–15.9)	12.7 (10.1–15.6)	.637
Child Pugh				
Class B (n, %)	142 (89)	50 (94)	92 (86)	.182
Class C (n, %)	18 (11)	3 (6)	15 (14)	.182
Bilirubin (μmol/L)	16 (10–25)	16 (10–23)	18 (11–28)	.166
Creatinine (μmol/L)	107 (78–147)	123 (98–161)	99 (74–129)	.001
Creatinine > 133 μmol/L (n, %)	50 (31)	24 (45)	26 (24)	.008
INR	1.27 (1.17–1.41)	1.23 (1.12–1.39)	1.33 (1.18–1.43)	.033
Platelets (10^3^/μL)	123 (85–179)	128 (101–177)	122 (80–179)	.319
Sodium (mmol/L)	135 (131–138)	136 (130–138)	135 (131–138)	.713
Cholinesterase (kU/L)	2.28 (1.67–2.97)	2.25 (1.82–2,97)	2.29 (1.62–2.97)	.755
Albumin (g/L)	28 (24–31)	30 (25–32)	28 (24–31)	.137
AST (U/L)	43 (33–56)	46 (35–60)	41 (30–54)	.115
ALT (U/L)	22 (16–35)	25 (19–40)	21 (14–33)	.042
AP (U/L)	132 (89–181)	135 (100–189)	125 (89–179)	.175
γ-GT (U/L)	135 (69–238)	146 (78–270)	122 (67–195)	.198
White blood cells (10^3^/μL)	5.8 (4.4–8.5)	5.5 (4.2–8.2)	6.0 (4.4–8.6)	.418
Haemoglobin (g/dL)	9.6 (8.7–11.7)	10.0 (8.9–11.9)	9.5 (8.6–11.2)	.215
History of SBP (n, %)	70 (44)	24 (46)	46 (43)	.706

* in 4 patients the etiology of cirrhosis is both, alcohol and viral, therefore the summation of percentages results in >100% in these columns.

Mann-Whitney-U-Test was used for continuous variables, chi-squared test or Fisher’s exact test for categorical variables. Shown is median with IQR or numbers with percentages. Abbreviations: PSG: portosystemic pressure gradient; NASH: non-alcoholic steatohepatitis; MELD: model for end-stage liver disease; CHE: cholinesterase; INR: international normalized ratio; AST: aspartate aminotransferase; ALT: alanine aminotransferase; AP: alkaline phosphatase; gGT: gamma glutamyl transferase; SBP: spontaneous bacterial peritonitis

### Periinterventional course and ascites control after TIPS insertion

The median duration of hospital stay after TIPS placement was 7 days (IQR_25-75_: 5–11, Table 2). Of note, in older patients hospital stay was significantly longer as compared to younger patients (8 vs. 6 days; p = 0.030). Regarding the periinterventional course after TIPS insertion, elderly patients suffered from significantly more ACLF episodes (21% vs. 9%; p = 0.044) as well as numerically more infections during the hospital stay after TIPS placement (26% vs. 17%; p = 0.153). In contrast, no difference was observed in terms of HE (p = 0.818 for HE grade 1–4 and p = 1.000 for severe HE) or AKI (p = 0.390 for any AKI and p = 0.513 for severe AKI). Creatinine level decreased from the time of TIPS insertion to the day of hospital demission in 74% of the patients (n = 119). A creatinine decrease was observed in 81% (n = 43) of the patients ≥65 years and in 71% (n = 76) of the younger patients (p = 0.159). Sufficient ascites control after 3 to 6 months after TIPS insertion was achieved in 68% of the patients. Of note, ascites persisted in a significantly higher proportion of younger patients as compared to the elderly population (37% vs. 12%; p = 0.048).

**Table 2:** Periinterventional course during hospital stay after TIPS insertion.

	All patients	≥ 65 years	< 65 years	*P* value
Patients (n, %)	160 (100)	53 (33)	107 (67)	
Hospital stay after TIPS (days)	7 (5–11)	8 (5–18)	6 (4–9)	.030
Infections (n, %)	32 (20)	14 (26)	18 (17)	.153
HE (grade 1–4) (n, %)	25 (16)	9 (17)	16 (15)	.818
Severe HE (grade 3–4) (n, %)	5 (3)	2 (4)	3 (3)	1.000
AKI (grade 1–3) (n, %)	33 (21)	13 (25)	20 (19)	.390
Severe AKI (grade 2–3) (n, %)	12 (8)	5 (9)	7 (7)	.513
ACLF (grade 1–3) (n, %)	21 (13)	11 (21)	10 (9)	.044
Creatinine decrease (n, %)*	119 (74)	43 (81)	76 (71)	.159

* Creatinine at TIPS insertion > creatinine at demission. Mann-Whitney-U-Test was used for continuous variables, chi-squared test or Fisher’s exact test for categorical variables. Shown is median with IQR or numbers with percentages. Abbreviations: HE: hepatic encephalopathy; AKI: acute kidney injury; ACLF: acute-on-chronic liver failure

### Impact of age on survival of patients after receiving a TIPS for refractory ascites

Overall, 31 patients (19%) died within the first year after TIPS placement. 19 of these patients were <65 years (corresponding to 18% of the patients <65 years) and 12 patients were ≥65 years (corresponding to 23% of the patients ≥65 years). Proportion of patients in whom death was primarily related to impaired liver function was higher among younger compared to the elderly patients (55% vs. 18% and 32% vs. 17% of death after 90 days and 1 year, respectively; S2 Table).

**Fig 2:**
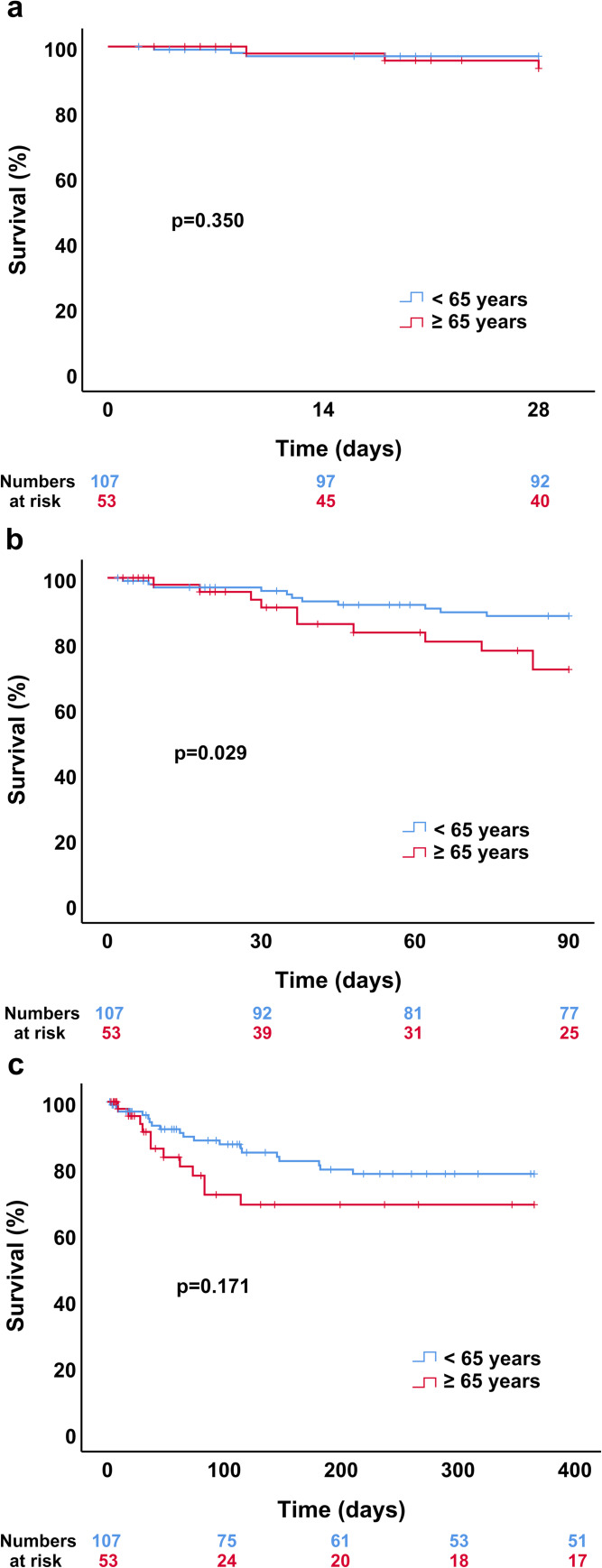
Survival of TIPS patients in dependence of age (<65 years vs. ≥65 years). Shown is survival (a) 28 days, (b) 90 days and (c) 1 year after TIPS insertion. *p*-values were obtained using the log-rank test (Breslow test: see text). A value of p<0.05 was considered statistically significant.

Mortality 28 days after TIPS placement was similar between patients <65 years and ≥65 years (p = 0.350; log-rank test; Fig 2A). In contrast, survival of younger patients was significantly higher at 90 days (p = 0.029; log-rank test; Fig 2B) and still showed a tendency towards longer survival 1 year after TIPS insertion (p = 0.171; log-rank test; Fig 2C). In order to particularly emphasize early occurring events, the Breslow test was performed in addition. This test confirmed the results obtained by the log-rank test: p = 0.405 for 28-day survival, p = 0.043 for 90-day survival and p = 0.134 for 1-year survival.

Of note, there was no difference in 1-year survival between ‘medium old’ (65–74 years) and ‘very old’ patients (≥75 years). However, this analysis was limited by the small number of patients in both groups (S2 Fig).

When adjusting for the degree of portal hypertension (PSG), sex, severity of liver disease (MELD) and etiology of cirrhosis, age ≥65 years remained an independent predictor for 90-day mortality (HR: 2.58; p = 0.028; Table 3B), while it was not linked to 28-day as well as 1-year mortality (Table 3A and 3C). The MELD score was associated with 90-day survival (HR: 1.20; p<0.001) as well as with 28-day survival (HR: 1.28; p<0.001) and 1-year survival (HR: 1.18; p<0.001), while sex, degree of portal hypertension and etiology of cirrhosis were no significant predictors. Within the subgroup of patients ≥65 years no parameter was independently associated with 28-day, 90-day and 1-year survival (S3 Table).

**Table 3:** A. Uni- and multivariate Cox regression analyzing risk factors for 28-day survival. B. Uni- and multivariate Cox regression analyzing risk factors for 90-day survival. C. Uni- and multivariate Cox regression analyzing risk factors for 1-year survival.

**A**						
**Risk factor for**	Univariate			**Multivariate HR**		
**28-day mortality**	**HR**	**95% CI**	***P***	**HR**	**95% CI**	***P***
Age ≥65 years	2.106	0.425–10.437	0.362			
PSG before TIPS (mmHg)	1.068	0.918–1.242	0.394			
MELD	1.282	1.137–1.446	< .001	1.281	1.136–1.445	< .001
Sex*	0.767	0.155–3.802	0.745			
Alcohol-related liver disease^#^	0.346	0.063–1.891	0.221			
**B**						
**Risk factor for**	**Univariate**			**Multivariate**		
**90-day mortality**	**HR**	**95% CI**	***P***	**HR**	**95% CI**	***P***
Age ≥65 years	2.462	1.066–5.687	0.035	2.577	1.106–6.001	0.028
PSG before TIPS (mmHg)	1.038	0.957–1.126	0.372			
MELD	1.189	1.098–1.288	< .001	1.196	1.104–1.296	< .001
Sex*	0.633	0.273–1.464	0.285			
Alcohol-related liver disease^#^	0.470	0.201–1.101	0.082			
**C**						
**Risk factor for**	** **	**Univariate HR**		** **	**Multivariate HR**	
**1-year mortality**		**95% CI**	***P***		**95% CI**	***P***
Age ≥65 years	1.649	0.799–3.403	0.176			
PSG before TIPS (mmHg)	1.041	0.974–1.112	0.233			
MELD	1.178	1.097–1.265	< .001	1.178	1.098–1.265	< .001
Sex*	0.701	0.346–1.417	0.322			
Alcohol-related liver disease^#^	0.627	0.310–1.268	0.194			

* female = reference.

^#^ other etiology than alcohol-related liver disease = reference.

All parameters tested in the univariate analysis were included in the multivariate model. Abbreviations: PSG: portosystemic pressure gradient; MELD: model for end-stage liver disease

### Comparison of TIPS vs. paracentesis in elderly patients with ascites

**Fig 3:**
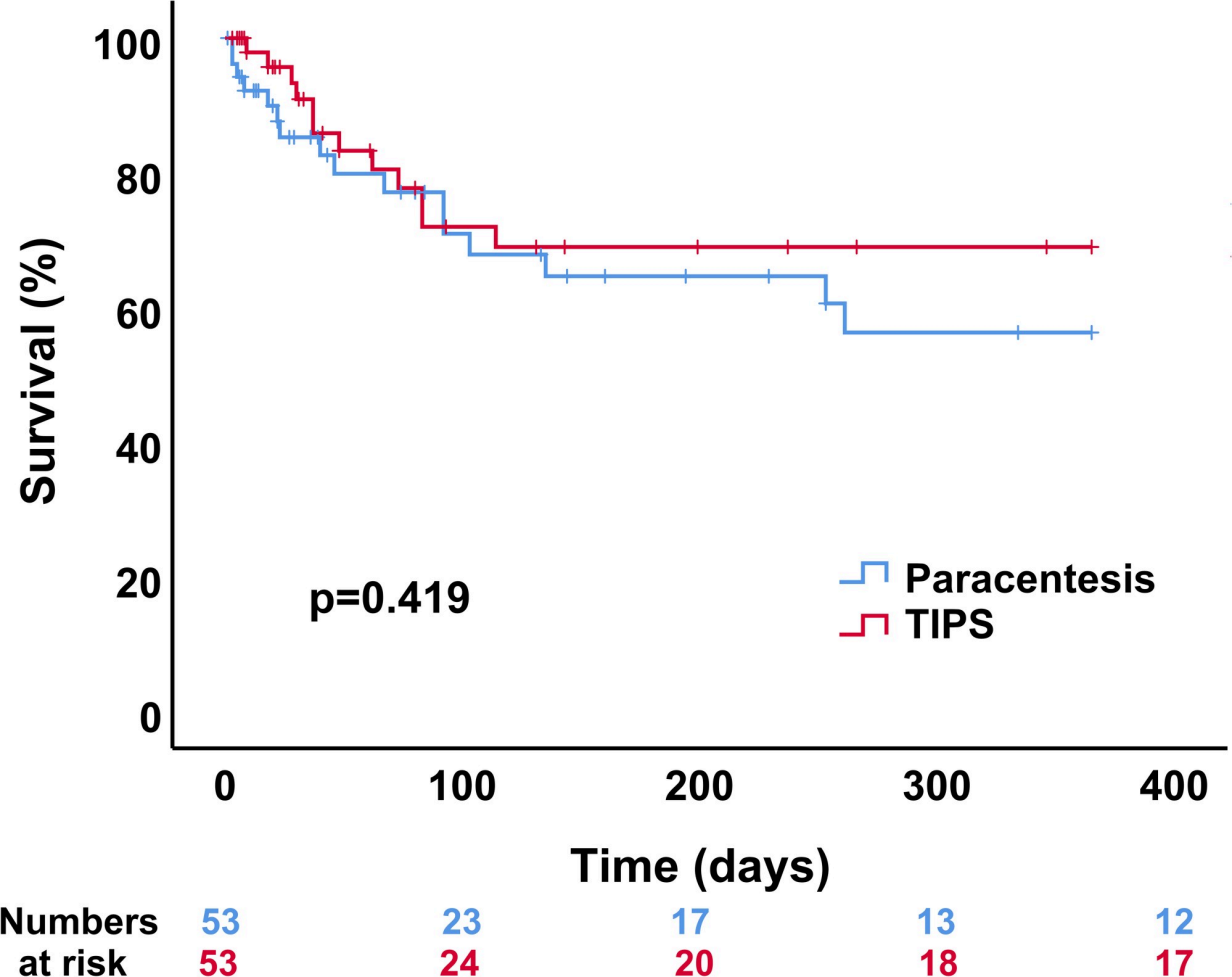
Comparison of 1-year survival between elderly cirrhotic TIPS patients and patients treated with paracentesis. The *p*-value was obtained using a stratified log-rank test and p<0.05 was considered statistically significant.

In order to appropriately compare survival of elderly TIPS patients with elderly patients treated with paracentesis, an 1:1 propensity score matching was conducted. The matching procedure resulted in satisfying balance between the 53 pairs as visually displayed in the respective Jitter and line plots (S1 Fig). Besides, the matching yielded absolute SMDs for the matching covariates, which were <0.25 representing no large imbalance (S1 Table). No significant differences were observed in any of the matching covariates after the matching procedure (Table 4). Of note, the MELD scores, supposed to represent the most important confounder, were notably balanced between the groups (12.6 vs. 13.0; p = 0.815). Subsequent comparison of survival between the matched elderly TIPS and paracentesis cohort revealed no significant difference in 1-year survival between patients treated with either TIPS or paracentesis (p = 0.419; Fig 3). Furthermore, mortality after 28 and 90 days after TIPS placement or first paracentesis was also similar (p = 0.196 and p = 0.808; S3 Fig).

**Table 4:** Comparison of baseline characteristics between matched elderly TIPS patients and patients treated with paracentesis.

Paired	All patients	TIPS	Paracentesis	*P* value
Patients (n, %)	106 (100)	53 (50)	53 (50)	
MELD	12.8 (10.4–15.8)	12.6 (10.8–15.9)	13.0 (10.4–15.7)	.815
Bilirubin (μmol/L)	16 (10–24)	16 (10–23)	16 (10–26)	.470
Age (years)	70 (67–75)	70 (68–75)	69 (67–75)	.480
Sex (male/female)	65 (61)/ 41 (39)	30 (57)/ 23 (43)	35 (66)/ 18 (34)	.472
Platelets (10^3^/μL)	125 (87–182)	128 (101–177)	120 (78–197)	.972
Sodium (mmol/L)	136 (132–138)	136 (130–138)	137 (132–139)	.226

Wilcoxon signed-rank test was used for continuous variables, McNemar test for categorical variables. Shown is median with IQR or numbers with percentages. Abbreviations: MELD: model for end-stage liver disease

## Discussion

When ascites becomes refractory the patients’ prognosis is dramatically aggravated [29]. By direct reduction of the portal venous pressure gradient TIPS represents a powerful tool for patients suffering from RA. However, a careful selection of patients is crucial. So far, the data on the safety and applicability of TIPS in the elderly population have been scarce and controversial. In the present study we addressed this clinically important topic and could demonstrate that TIPS placement in patients ≥65 years seems to be overall well feasible. However, elderly patients require special attention, in particular in the early phase after TIPS insertion, as we documented a more complicated periinterventional course and a significantly higher 90-day mortality. Of note, 1-year survival was not markedly impaired as compared to younger individuals and importantly, we showed for the first time that TIPS is indeed not inferior to paracentesis in the elderly cohort with RA.

Mortality in cirrhotic patients with RA is remarkably high irrespective of age. We hypothesize that the survival difference documented within the first three months after TIPS insertion and the more complicated periinterventional course might not only be attributable to the inherent effect of higher age, but also to a more challenging TIPS adaption process in older individuals. The higher proportion of death from non-liver-related causes in this time span may further emphasize the need for a careful assessment of comorbidities and general health status in the older population prior to TIPS. However, we were not able to identify any particular risk factor for mortality after TIPS among elderly patients in our study.

Remarkably, the role of age as a predictor for survival after TIPS has been controversially discussed in the current literature, so far: Age was identified as a predictor for post-TIPS mortality in some [30–37], but not in all studies [38–41]. This discordance could be ascribed to methodological dissimilarities between the different studies such as the particular age thresholds for the definition of the ‘elderly’ population, various sample sizes, the type of stent used or the TIPS indication (RA vs. variceal bleeding). For instance, we chose an age threshold of 65 years, because this is a widely accepted age limit for patients, who need a more careful evaluation before liver transplantation (LTx), since patients ≥65 years have been shown to have a higher mortality after LTx [42]. Therefore, patients ≥65 years are more often excluded from the only curative treatment option LTx. The same age limit of 65 years was deployed by Suraweera *et al*., who performed a propensity score matching between 30 elderly and 30 non-elderly TIPS patients and could find no difference in terms of 90-day survival [38]. However, the sample size in this study was very low and the patient population was more heterogeneous, since they included patients receiving a TIPS for either RA as well as for variceal bleeding. Bucsics *et al*. identified age as an independent predictor for post-TIPS mortality [37]. The strength of this study was that–in accordance with our study–only covered stent grafts were used. The study population analyzed by Syed *et al*. was particularly small including only 23 patients ≥65 years [43]. In this study, in which a control group of younger patients is missing, the authors concluded that TIPS is capable to control portal hypertension-related complications such as bleeding and ascites in elderly patients. The hitherto largest analysis evaluating TIPS in patients of higher age included 539 individuals, of whom 65 were ≥70 years old [30]. In concordance with our results, this group revealed age as well as MELD score as significant predictors for 90-day survival. Of note, no paracentesis control group was incorporated any of these studies.

Indeed, the majority of studies conducted to date investigated particular risk factors such as higher age with regard to the impact on outcome after TIPS insertion [30,31,38,43]. However, what is clinically at least equally important, is the comparison of the outcome between elderly TIPS patients and a non-TIPS control group. We particularly addressed this demand by including a propensity score matched control group of cirrhotic patients of higher age, who were managed with paracentesis. To our knowledge this is the first study, in which propensity score matching was performed to match elderly cirrhotic patients with RA treated with either TIPS or paracentesis. Of note, 1-year survival was not inferior among elderly patients if treated with TIPS instead of paracentesis. However, it is important to acknowledge that on the other hand we also did not document a significant survival benefit in the TIPS cohort. Moreover, the periinterventional course was slightly more complicated than in younger adults. Thus, if available, LTx should always be evaluated as first therapeutic option for RA also in the elderly patients, since this is the only curative treatment with sufficient evidence for prolonging the patients’ survival.

Another strength of our study is that only covered stent grafts were inserted in our patients, since in comparison to bare stents, coated TIPS were proven to result in improved graft patency as well as increased rates of relief from portal hypertension-related complications [44]. Moreover, only patients receiving a TIPS for RA were considered resulting in a homogeneous study population.

However, our investigation also has some limitations that need to be considered: First, the present study is non-randomized, which we tried to partly overcome by using the propensity score matching. Secondly, data assessment and analysis was done retrospectively based on the patients’ medical files. Furthermore, this study is a single-institution analysis, which on the one hand reduces interinstitutional variations, but on the other hand results in less generalizable conclusions. Moreover, the implications drawn from our study can only be transferred to patients receiving a TIPS for RA, since only these patients were included in our analysis. Future research should include a multicenter validation cohort. Furthermore, there might be an inherent selection bias regarding the allocation of patients to TIPS vs. paracentesis with fitter patients being referred to the TIPS group. However, we tried to minimize this bias by excluding patients fulfilling any TIPS contraindication and by adjusting the MELD scores and bilirubin levels between the groups using the propensity score matching approach.

In conclusion, our study indicates that TIPS placement in cirrhotic elderly patients with RA is generally safe, efficient and feasible. However, clinicians should be familiar with the fact that patients of higher age may require more caution and counseling about the risk and benefits of TIPS insertion. Therefore, TIPS creation should be pursued cautiously in older patients. However, if LTx is not an option, a higher age should not be considered as an absolute contraindication for TIPS, since the survival of elderly TIPS patients does not seem to be impaired as compared to older cirrhotic patients treated with paracentesis.

## Supporting information

S1 Dataset Minimal data set. (XLSX)

S1 Fig Visualization of model adequacy of the propensity score matching. Shown is (a) a Jitter plot illustrating the distribution of individual propensity scores and (b) a line plot displaying the absolute standardized mean differences (SMD) of each matching covariate.
(DOCX)

S2 Fig Comparison of 1-year survival after TIPS insertion between patients <65 years, 65–74 years (‘medium old’) and ≥75 years (‘very old’). The *p*-value was obtained using the log-rank test and p<0.05 was considered statistically significant.
(DOCX)

S3 Fig Comparison of survival between elderly cirrhotic TIPS patients and patients treated with paracentesis. Shown is (a) 28-day as well as (b) 90-day survival. *p*-values were obtained using a stratified log-rank test and p<0.05 was considered statistically significant.
(DOCX)

S1 Table Comparison of matching covariates between elderly TIPS patients and patients treated with paracentesis. Shown are means or numbers and standardized mean differences (SMD) before and after the matching procedure. Abbreviations: MELD: model for end-stage liver disease; SMD: standardized mean difference.
(DOCX)

S2 Table A. Causes of death of TIPS patients dying within 90 days after TIPS insertion. B. Causes of death of TIPS patients dying within 1 year after TIPS insertion.
(DOCX)

S3 Table Univariate Cox regression analyzing risk factors for (a) 28-day, (b) 90-day and (c) 1-year survival after TIPS insertion in the subgroup of patients ≥65 years.
(DOCX)

## Abbreviations

RA: refractory ascites
TIPS: transjugular intrahepatic portosystemic shunt
ACLF: acute-on-chronic liver failure
HE: hepatic encephalopathy
SBP: spontaneous bacterial peritonitis
AKI: acute kidney injury
EASL: European Association for the Study of the Liver
OPS: operation and procedure code
PSG: portosystemic pressure gradient
MELD: model for end-stage liver disease
IQR: interquartile range
SMD: standardized mean differences
INR: international normalized ratio
CHE: cholinesterase
AST: aspartate aminotransferase
ALT: alanine aminotransferase
AP: alkaline phosphatase
gGT: gamma glutamyl transferase
Hb: Hemoglobin
NASH: non-alcoholic steatohepatitis
HR: hazard ratio
CI: confidence interval
LTx: liver transplantation
