# Supplementary material for: Safety and feasibility of transjugular intrahepatic portosystemic shunt in elderly patients with liver cirrhosis and refractory ascites
Source: PLoS One. 2020 Jun 25;15(6):e0235199. doi: 10.1371/journal.pone.0235199 (PMC7316253; doi:10.1371/journal.pone.0235199)
Supplement: S1 Fig — Shown is (a) a Jitter plot illustrating the distribution of individual propensity scores and (b) a line plot displaying the absolute standardized mean differences (SMD) of each matching covariate. (DOCX) [file pone.0235199.s002.docx]

**S1 Fig.** **Visualization of model adequacy of the propensity score matching.**

Shown is (a) a Jitter plot illustrating the distribution of individual propensity scores and (b) a line plot displaying the absolute standardized mean differences (SMD) of each matching covariate.
