# Supplementary material for: Safety and feasibility of transjugular intrahepatic portosystemic shunt in elderly patients with liver cirrhosis and refractory ascites
Source: PLoS One. 2020 Jun 25;15(6):e0235199. doi: 10.1371/journal.pone.0235199 (PMC7316253; doi:10.1371/journal.pone.0235199)

**S2 Fig.** **Comparison of 1-year survival after TIPS insertion between patients <65 years, 65-74 years (‘medium old’) and ≥75 years (‘very old’).**

The *p*-value was obtained using the log-rank test and p<0.05 was considered statistically significant.


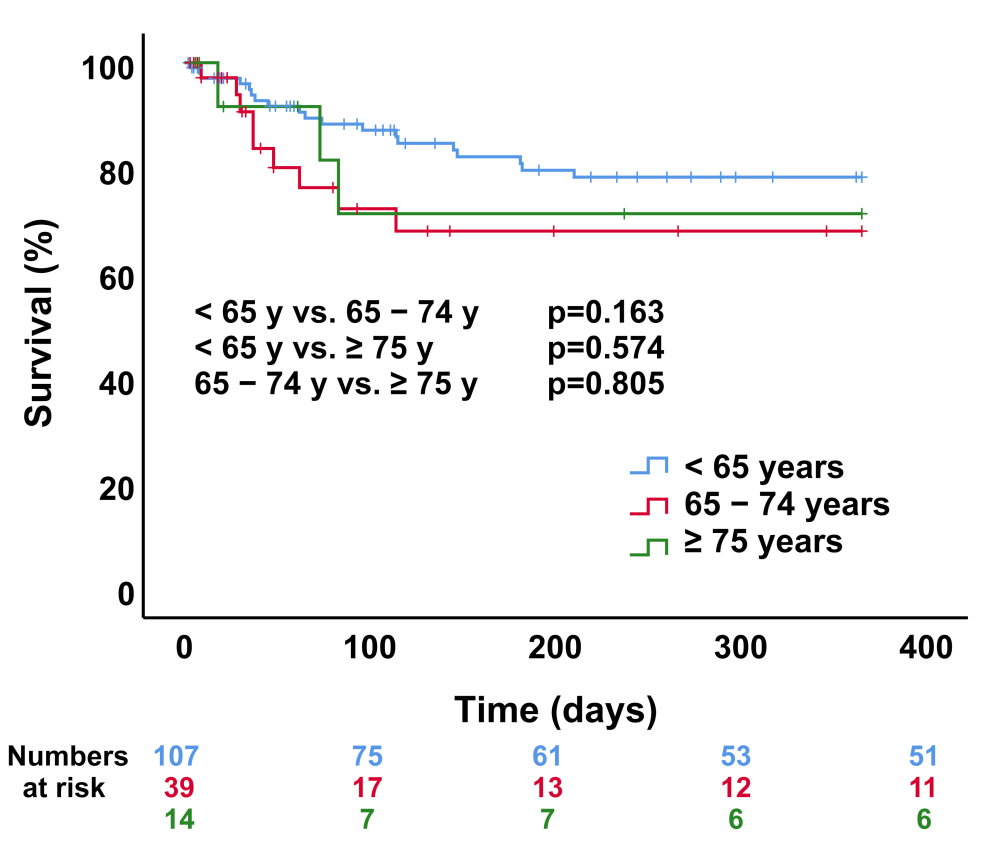

Supplement: S2 Fig — The p-value was obtained using the log-rank test and p<0.05 was considered statistically significant. (DOCX) [file pone.0235199.s003.docx]
