# Supplementary material for: Safety and feasibility of transjugular intrahepatic portosystemic shunt in elderly patients with liver cirrhosis and refractory ascites
Source: PLoS One. 2020 Jun 25;15(6):e0235199. doi: 10.1371/journal.pone.0235199 (PMC7316253; doi:10.1371/journal.pone.0235199)

**S3 Fig. Comparison of survival between elderly cirrhotic TIPS patients and patients treated with paracentesis.**

Shown is (a) 28-day as well as (b) 90-day survival. *p*-values were obtained using a stratified log-rank test and p<0.05 was considered statistically significant.


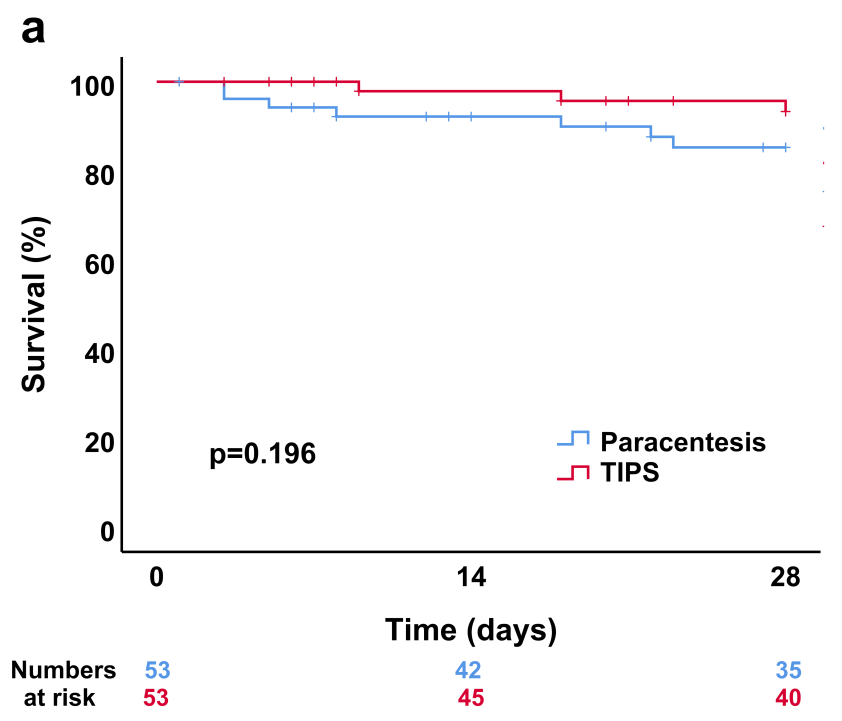


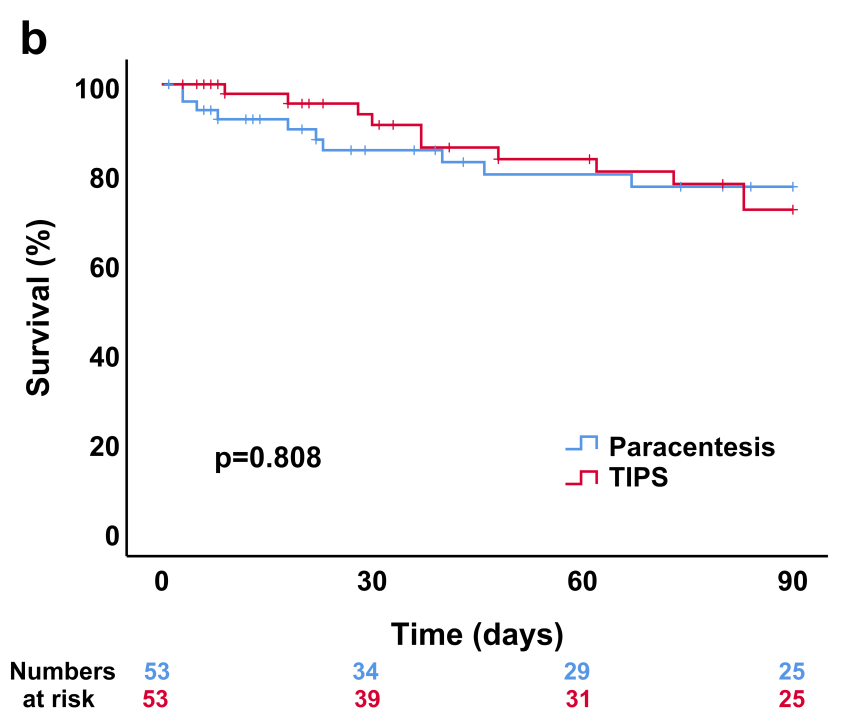

Supplement: S3 Fig — Shown is (a) 28-day as well as (b) 90-day survival. p-values were obtained using a stratified log-rank test and p<0.05 was considered statistically significant. (DOCX) [file pone.0235199.s004.docx]
