# Supplementary material for: Safety and feasibility of transjugular intrahepatic portosystemic shunt in elderly patients with liver cirrhosis and refractory ascites
Source: PLoS One. 2020 Jun 25;15(6):e0235199. doi: 10.1371/journal.pone.0235199 (PMC7316253; doi:10.1371/journal.pone.0235199)
Supplement: S1 Table — Shown are means or numbers and standardized mean differences (SMD) before and after the matching procedure. Abbreviations: MELD: model for end-stage liver disease; SMD: standardized mean difference. (DOCX) [file pone.0235199.s005.docx]

**S1 Table. Comparison of matching covariates between elderly TIPS patients and patients treated with paracentesis.**

| Covariates | Before matching TIPS | Paracentesis | SMD | After matching TIPS | Paracentesis | SMD |
| --- | --- | --- | --- | --- | --- | --- |
| Patients (n) | 53 | 85 |  | 53 | 53 |  |
| Propensity score | 0.506 | 0.308 | 1.181 | 0.506 | 0.438 | 0.404 |
| MELD | 13.4 | 15.1 | -0.392 | 13.4 | 13.3 | 0.010 |
| Bilirubin (µmol/L) | 17 | 44 | -2.550 | 17 | 19 | -0.166 |
| Age (years) | 71.3 | 70.4 | 0.202 | 71.3 | 70.9 | 0.086 |
| Sex (male/ female) | 30 (57)/23 (43) | 57 (67)/28 (33) | 0.209 | 30 (57)/23 (43) | 35 (66)/18 (34) | 0.189 |
| Platelets (10^3^/µL) | 145 | 148 | -0.049 | 145 | 145 | -0.003 |
| Sodium (mmol/L) | 134 | 136 | -0.275 | 134 | 136 | -0.231 |

Shown are means or numbers and standardized mean differences (SMD) before and after the matching procedure. Abbreviations: MELD: model for end-stage liver disease; SMD: standardized mean difference
