# Supplementary material for: Safety and feasibility of transjugular intrahepatic portosystemic shunt in elderly patients with liver cirrhosis and refractory ascites
Source: PLoS One. 2020 Jun 25;15(6):e0235199. doi: 10.1371/journal.pone.0235199 (PMC7316253; doi:10.1371/journal.pone.0235199)
Supplement: S2 Table — A. Causes of death of TIPS patients dying within 90 days after TIPS insertion. B. Causes of death of TIPS patients dying within 1 year after TIPS insertion. (DOCX) [file pone.0235199.s006.docx]

**S2a Table. Causes of death of TIPS patients dying within 90 days after TIPS insertion.**

| Cause of death | All patients | ≥ 65 years (n, %) | < 65 years (n, %) |
| --- | --- | --- | --- |
| Liver-related | 8 (36) | 2 (18) | 6 (55) |
| Cardiac decompensation | 1 (5) | 1 (9) | 0 (0) |
| Infection | 3 (14) | 2 (18) | 1 (9) |
| Other/unknown | 10 (45) | 6 (55) | 4 (36) |
| Total | **22 (100)** | **11 (100)** | **11 (100)** |

**S2b Table. Causes of death of TIPS patients dying within 1 year after TIPS insertion.**

| Cause of death | All patients | ≥ 65 years (n, %) | < 65 years (n, %) |
| --- | --- | --- | --- |
| Liver-related | 8 (26) | 2 (17) | 6 (32) |
| Cardiac decompensation | 2 (6) | 1 (8) | 1 (5) |
| Infection | 4 (13) | 2 (17) | 2 (10) |
| Other/unknown | 17 (55) | 7 (58) | 10 (53) |
| Total | **31 (100)** | **12 (100)** | **19 (100)** |

Shown are numbers and percentages in parenthesis.
