# Supplementary material for: Safety and feasibility of transjugular intrahepatic portosystemic shunt in elderly patients with liver cirrhosis and refractory ascites
Source: PLoS One. 2020 Jun 25;15(6):e0235199. doi: 10.1371/journal.pone.0235199 (PMC7316253; doi:10.1371/journal.pone.0235199)
Supplement: S3 Table — Univariate Cox regression analyzing risk factors for (a) 28-day, (b) 90-day and (c) 1-year survival after TIPS insertion in the subgroup of patients ≥65 years. (DOCX) [file pone.0235199.s007.docx]

**S3a Table. Univariate Cox regression analyzing risk factors for 28-day survival after TIPS insertion in the subgroup of patients ≥65 years.**

| Risk factor | Univariate HR | 95% CI | *P* |
| --- | --- | --- | --- |
| Age (years) | 1.173 | 0.919-1.496 | .199 |
| Sex* | 0.577 | 0.081-4.095 | .582 |
| PSG before TIPS (mmHg) | 1.089 | 0.910-1.304 | .351 |
| MELD | 1.144 | 0.979-1.336 | .091 |
| Bilirubin (µmol/L) | 1.026 | 0.947-1.110 | .531 |
| Creatinine (µmol/L) | 1.007 | 0.998-1.017 | .141 |
| INR | 4.259 | 0.036-499.491 | .551 |
| Platelets (10^3^/µL) | 0.992 | 0.972-1.013 | .454 |
| Sodium (mmol/L) | 1.229 | 0.913-1.652 | .174 |

* female = reference.

**S3b Table. Univariate Cox regression analyzing risk factors for 90-day survival after TIPS insertion in the subgroup of patients ≥65 years.**

| Risk factor | Univariate HR | 95% CI | *P* |
| --- | --- | --- | --- |
| Age (years) | 1.024 | 0.885-1.183 | .753 |
| Sex* | 0.795 | 0.242-2.606 | .704 |
| PSG before TIPS (mmHg) | 1.031 | 0.936-1.136 | .536 |
| MELD | 1.088 | 0.942-1.256 | .250 |
| Bilirubin (µmol/L) | 1.021 | 0.972-1.072 | .410 |
| Creatinine (µmol/L) | 1.004 | 0.995-1.013 | .369 |
| INR | 2.643 | 0.140-49.847 | .517 |
| Platelets (10^3^/µL) | 1.001 | 0.992-1.011 | .784 |
| Sodium (mmol/L) | 1.050 | 0.921-1.197 | .467 |

* female = reference.

**S3c Table. Univariate Cox regression analyzing risk factors for 1-year survival after TIPS insertion in the subgroup of patients ≥65 years.**

| Risk factor | Univariate HR | 95% CI | *P* |
| --- | --- | --- | --- |
| Age (years) | 1.010 | 0.879-1.160 | .891 |
| Sex* | 0.906 | 0.287-2.860 | .867 |
| PSG before TIPS (mmHg) | 1.017 | 0.925-1.118 | .724 |
| MELD | 1.079 | 0.935-1.244 | .300 |
| Bilirubin (µmol/L) | 1.015 | 0.967-1.066 | .544 |
| Creatinine (µmol/L) | 1.004 | 0.996-1.013 | .330 |
| INR | 1.659 | 0.091-30.406 | .733 |
| Platelets (10^3^/µL) | 1.003 | 0.994-1.011 | .553 |
| Sodium (mmol/L) | 1.073 | 0.941-1.224 | .291 |

* female = reference.

Abbreviations: PSG: portosystemic pressure gradient; MELD: model for end-stage liver disease; INR: international normalized ratio
